# Enhancing prognostic accuracy in PMBCL: semiquantitative analysis of interim PET/CT scans

**DOI:** 10.1038/s41598-025-18649-9

**Published:** 2025-10-07

**Authors:** V. Hanáčková, K. Polgárová, L. Henzlová, D. Zogala, A. Obr, M. Trněný, T. Papajík, V. Procházka

**Affiliations:** 1 Department of Haemato-oncology, Faculty of Medicine and Dentistry, Palacký University and University Hospital Olomouc, Olomouc, Czech Republic; 2 Department of Nuclear Medicine, Faculty of Medicine and Dentistry, Palacký University and University Hospital Olomouc, Olomouc, Czech Republic; 3 First Department of Internal Medicine-Hematology, First Faculty of Medicine, Charles University and the General University Hospital in Prague, Prague, Czech Republic; 4 Institute of Nuclear Medicine, First Faculty of Medicine, Charles University and the General University Hospital in Prague, Prague, Czech Republic

**Keywords:** Primary mediastinal large b-cell lymphoma, PET/CT, Radiomics, Interim PET, Quantitative metrics, Event-free survival, Health care, Haematological cancer, Haematological cancer

## Abstract

Primary mediastinal large B-cell lymphoma (PMBCL) is a rare, aggressive lymphoma affecting young adults. Interim PET/CT (iPET/CT) scans are used to assess treatment response, but the positive predictive value of standard Deauville score remains limited. This retrospective multicenter study analyzed 116 PMBCL patients treated with anthracycline-based chemoimmunotherapy, focusing on 90 patients with high quality iPET/CT. Semiquantitative radiomics metrics, including changes in maximum standardized uptake value (dSUVmax), metabolic tumor volume (dMTV), and total lesion glycolysis (dTLG), were assessed alongside event-free survival (EFS). All interim and final PET/CT scans were independently reviewed by two nuclear medicine physicians blinded to outcomes. Among the 90 patients, 62 (68.9%) were iPET-positive (Deauville scores 4–5). Event-free survival (EFS) at 3 years was significantly higher in iPET-negative patients compared to iPET-positive patients (75% vs. 29%; *p* < 0.01). Radiomics analysis demonstrated that dSUVmax, dMTV, and dTLG provided superior predictive accuracy for EFS. Values below optimized cut-off thresholds demonstrated significantly better outcomes (e.g., 3-y EFS: 77.8% for dSUVmax ≥ 80% vs. 11.1% for dSUVmax < 80%, *p* < 0.01). Radiomics-based metrics outperformed visual iPET/CT assessment in identifying high-risk patients, underscoring their potential in guiding treatment. Future research should integrate radiomics with clinical factors to enhance PET-guided treatment strategies.

## Introduction

Primary mediastinal large B-cell lymphoma (PMBCL) is an aggressive and rare subtype of non-Hodgkin lymphoma. The disease represents 2–4% of all non-hodgkin lymphomas (NHL) and primarily affects younger individuals and more commonly women. PMBCL has distinct clinicopathological and molecular characteristics^1^. Symptoms of the disease are characterized by rapid progression of anterior mediastinal mass, including cough, dyspnea or even superior vena cava syndrome, pleural and pericardial effusion may be seen. Pathological and molecular profile of the disease overlaps with classic Hodgkin lymphoma even though disease is considered as a separated subtype^2,3^. Morphologically, the tumor is composed of diffuse proliferation of medium to large B lymphocytes and from an immunohistochemical perspective, PMBCL cells express B-cell antigens, and in approximately 80% of cases CD30 expression is present^4^. Genetic alterations in PMBCL differ from the genetic profile of diffuse large B-cell lymphoma and more closely resemble specific genetic profile of cHL. Currently, standard anthracycline-based immunochemotherapy (R-CHOP, DA-EPOCH-R) is used in treatment, along with radiotherapy or autologous peripheral stem cell transplantation consolidation on residual tumor mass. The 5-year progression-free survival rate ranges between 70 and 80% ^5^, yet some patients experience early relapse within one year of completing therapy, with a poor prognosis for these patients^6–8^. Early identification of poor responders is crucial for avoiding unnecessary chemotherapy, enabling more targeted consolidation strategies, and identifying candidates for alternative therapy such as CAR-T cells, PD1 inhibitors or brentuximab-vedotin^9–11^.

In diagnosis, staging, and monitoring treatment response, positron emission tomography combined with computed tomography (PET/CT) using 18 F-fluorodeoxyglucose (FDG) is widely utilized^12^. The uptake intensity of 18 F-FDG in the tumor mass on PET/CT reflects glucose metabolism in tumor cells as well as in the microenvironment, including processes such as necrosis or apoptosis, and determines the metabolic heterogeneity of the tumor. This variability can potentially reflect the tumor’s aggressiveness, as some studies conducted on solid tumors have already demonstrated^13,14^. The basic visual assessment of FDG PET/CT scans uses the 5-point Deauville scale. Recent studies verified an impact of the pre-treatment PET and end of induction PET radiomics features in determining prognosis^15,16^. The studies evaluating the utility of the PET during the therapy (interim) are scarce, based on limited cohorts, and have shown conflicting results^17–19^. Previous studies primarily focused on visual assessment using the Deauville score. Our study uniquely contributes to the field by incorporating multiple semiquantitative PET parameters. We aimed to analyze interim PET/CT scans in the large cohort of the PMBCL patients from the two academic institutions, treated with chemo-immunotherapy, whose scans have been independently centrally reviewed.

## Methods

### Ethical statement

The study was conducted on behalf of the Czech Hodgkin Lymphoma Study Group (CHSG; NCT06263530). The study was approved by the respective local Ethics Committees and conducted in accordance with the Declaration of Helsinki. According to ethical policies of the University Hospital Olomouc and General Hospital Prague, clinical data can be analyzed and used under the precondition of without revealing the identity of patients. All patients provided informed written consent for anonymous processing of their clinical data.

### Patient population

The study comprised patients with diagnosis of PMBCL enrolled into prospectively operated Czech Lymphoma Study Group (CLSG) registry from the two academic centers in Czech Republic (University Hospital Olomouc and General University Hospital Prague) between 1 January 2005 and 31 December 2022.

All patients were treated with anthracycline-based therapy with or without autologous stem cell transplantation or consolidation radiotherapy. All data including the primary therapy protocol intent and the reason for the therapy adaptation were reviewed by treating physicians and the database was locked by the 15 September 2024.

**Fig. 1:**
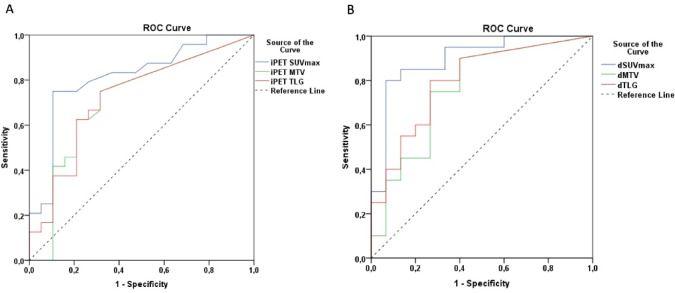
Receiver-operating curve (ROC) analysis for predicting optimal cut off value for metric PET parameters: (**A**) ROC analysis for iPET SUVmax, MTV and TLG, (**B**) ROC analysis for iPET dSUVmax, dMTV and dTLG.

From a total of 116 cases identified, 115 patients were confirmed to have diagnosis of PMBCL according to the central pathology review. Another 25 patients were excluded from the analysis for imaging technical issues (poor quality of scans, different scanning protocols, *n* = 17) or patients were excluded due to missing iPET/CT scan (*n* = 8). (Fig. 1). Finally, we identified 90 patients with high-quality interim and final PET/CT performed after the second (50%) or third (45.6%) cycle of immunochemotherapy. Only four patients had interim PET/CT after the fourth cycle of immunochemotherapy.

All the patients were treated with standard rituximab-anthracycline-based chemotherapy, 29 patients (32.3%) were treated with R-CHOP (rituximab, prednisone, cyclophosphamide, doxorubicine, vincristine), 17 patients (18.9%) with regimen containing etoposide such as R-CHOEP (rituximab, cyclophosphamide, doxorubicine, vincristine, etoposide, prednisone) or R-PACEBO (rituximab, prednisone, cyclophosphamide, doxorubicine, vincristine, etoposide, bleomycine), 44 patients (48.9%) were treated with intensive R-SQ chemotherapy protocol or R-MegaCHOP/ESHAP regimen^20,21^. Intensive R-SQ protocol consisted of six cycles of chemotherapy: four R-PACEBO (rituximab, prednisone, doxorubicin, cyclophosphamide, etoposide, bleomycin, vincristine) cycles, one cycle of an ifosfamide and methotrexate-based regimen (R-IVAM, rituximab, cytarabine, ifosfamide, etoposide, methotrexate), one cycle of a priming regimen with high-dose cytosine arabinoside (R-HAM, rituximab, cytosine arabinoside, mitoxantrone) and eventually with BEAM regimen and ASCT. In our cohort, 34 (37.8%) patients were consolidated by radiotherapy and 39 patients (43.3%) underwent ASCT. Both treatment modalities underwent 7 of these patients (7.8%). The treatment followed PMBCL guidelines and local clinical practices. All methods were carried out in accordance with relevant guidelines and regulations and experimental protocols were approved by an institutional committee.

### Intention-to-treat therapy change

In our cohort, we identified patients in whom the interim PET/CT results led to a change in the intention-to-treat (ITT) therapy. A change of ITT therapy was defined as an intensification of treatment due to the inefficacy of the current regimen, based on positive iPET/CT findings, including the administration of intensive chemotherapy protocol, the incorporation of ASCT, or radiotherapy consolidation.

### PET imaging protocol

All patients fasted for at least 6 h prior the scan and their blood glucose levels were below 5.8 mmol/L before the^18^F-FDG injection (200 MBq / 70 kg patient). Injected activities were adjusted to patients’ weight following the EANM procedure guidelines^22^. The delay between injection and PET/CT imaging procedure was 60 ± 5 min. In the first center, a base of the skull to mid-thighs scans were acquired using Siemens Biograph mCT40 (Siemens Healthcare GmbH, Erlangen, Germany) with the following acquisition and reconstruction parameters: CT – 120 kV (CARE kV off), eff. mAs 120 (CARE Dose 4D on), SAFIRE (strength 3), I30f convolution kernel, reconstructed slice thickness 3 mm, reconstruction increment 1.5 mm; PET – 3 min / bed position, 256 × 256 reconstruction matrix, 3 iterations, 25 subsets, PSF + TOF, 5 mm Gaussian post-reconstruction filter, scatter, randoms and attenuation corrections applied. The second center used other PET/CT system (Discovery 690, GE Healthcare, Milwaukee, WI, USA). The administered dose of FDG was 4.6 MBq per kilogram of body weight, the minimum postinjection resting time was 60 min (84 ± 14, 58–119 min). The PET images were reconstructed using the OSEM algorithm including time-of-flight and resolution recovery techniques with the following parameters: matrix 256 × 256 pixels, 3 iterations, 32 subsets, and 6.0 mm postfilter. CT data was used for attenuation correction of PET images. CT acquisition was performed prior to PET with the following parameters: peak tube voltage, 120–140 kV; tube current, 30–210 mA; pitch, 1.0; rotation time, 0.7 s. Contrast enhancement with 100 ml of iodinated contrast agent (Iomeron 400, Bracco Ltd., UK) was used in case of absence of contraindications.

Both PET systems, utilizing the acquisition and reconstruction protocols listed above, met the EARL v2 accreditation criteria. As part of the accreditation process, PET/CT image reconstruction and interpretation were harmonized using quantitative and image-quality phantom scans, followed by adjustment of recovery coefficient curves to ensure compliance with the accreditation acceptance range. This standardization reduces inter-institutional variability in SUV measurements, ensuring that reconstructed images and quantitative results obtained from different EARL v2 accredited systems can be compared, exchanged, and combined — regardless of the institution of origin or the software platform used for evaluation. Quantitative analysis was based on SUVmax and the derived metrics of MTV and TLG, using a 41% SUVmax threshold. This thresholding method is supported by EANM procedure guidelines^22^. All image analyses were performed using the syngo.via platform (Siemens Healthcare GmbH, Erlangen, Germany) with the MM Oncology package using the above mentioned threshold. All the interim and final PET/CT scans were independently re-evaluated by two nuclear medicine physicians blinded to clinical outcomes. Treatment response was assessed according to the Lugano 2014 classification. Interim PET/CT scans were evaluated using the Deauville 5-point scale, with Deauville 1–3 considered as PET-negative and Deauville 4–5 considered as PET-positive.

### Statistical analysis

The data were analyzed using the Statistical Package for the Social Sciences (IBM SPSS Statistics for Windows, Version 21.0. Armonk, NY: IBM Corp.). The differences between the iPET/CT positive and iPET/CT negative groups were analyzed using the chi-squared and Mann‐Whitney *U* tests for qualitative and quantitative variables, respectively. The Kaplan‐Meier method was used to estimate the survival probabilities. The log‐rank test was used to compare difference in survival between patient subgroups. The significance level was set to *P* = 0.05; two‐tailed tests were used in all calculations.

Overall survival (OS) was defined as the time from the diagnosis (OS) to the date of the last follow-up examination (censored) or the date of death (event) from any cause. Progression‐free survival (PFS) was defined as the time from diagnosis to relapse, progression, or death from any cause. Event free survival (EFS) was defined as relapse, progression, death of any cause or therapy protocol change due to the ineffectivity in patients with positive iPET/CT (Table 1). Receiver-operating curve (ROC) analysis was done to find optimal cut off value for metric PET parameters. (Fig. 1)

**Table 1:** Types of event free survival events in analyzed cohort.

EFS event	All patients, *n* = 90	iPET negative patients, *n* = 28	iPET positive patients, *n* = 62
Total of EFS events	53	9	44
Change of therapy*	41 (77.4%)	5 (55.6%)	36 (81.8%)
Relapse/progression	10 (18.9%)	3 (33.3%)	7 (15.9%)
Death	2 (3.8%)	1 (11.1%)	1 (2.3%)

*an intensification of treatment due to the inefficacy of the current regimen, including the administration of intensive chemotherapy protocol, the incorporation of ASCT, or radiotherapy consolidation.

No formal adjustment for multiple comparisons was performed, as this study was exploratory.

## Results

### Clinical features and characteristics

**Fig. 2:**
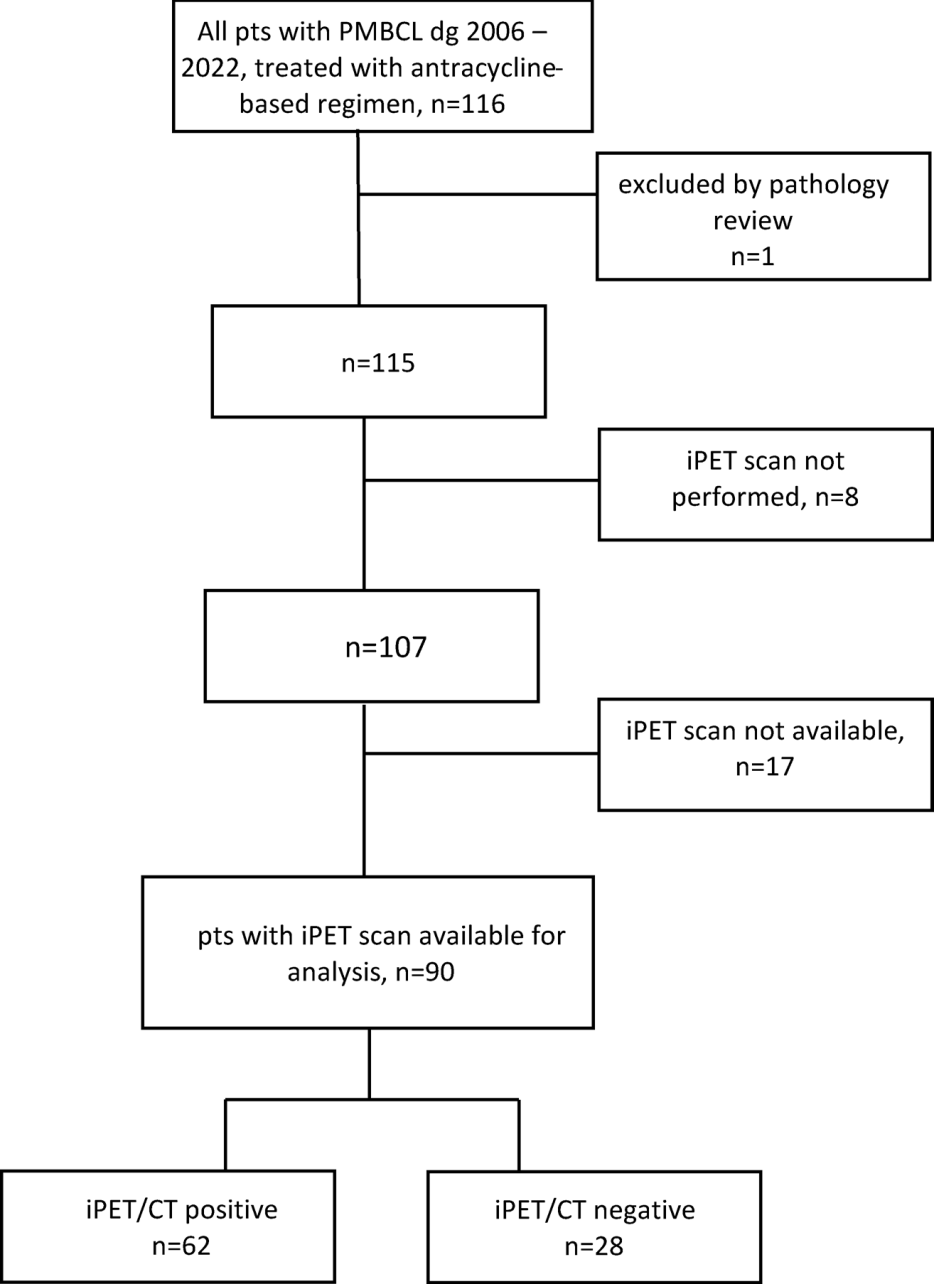
CONSORT flow diagram showing the patient selection process.

A comparison of baseline characteristics between the analyzed (*n* = 90) and excluded (*n* = 26) patients showed no significant differences. Among excluded patients, 65.4% were female and 34.6% male, compared to 66.7% and 33.3% in the analyzed cohort (*p* = 0.90). The mean age was 34 years in excluded patients and 37 years in analyzed patients (*p* = 0.45). Clinical stage I–II was present in 69.8% of excluded patients and 71.1% of analyzed patients, while stage III–IV was found in 30.% and 28.%, respectively (*p* = 0.75). Regarding prognostic risk, low-risk IPI (scores 1–2) was observed in 76.9% of excluded and 81.1% of analyzed patients, and high-risk IPI (≥ 3) in 23.1% and 18.9%, respectively (*p* = 0.56). These results indicate that the two cohorts did not differ meaningfully in terms of basic clinical characteristics. The analyzed cohort consisted of 90 out of 116 patients with a diagnosis of PMBCL with available interim PET/CT between 2005 and 2022 (Fig. 2). In the cohort, there were 30 men and 60 women with a median age of 37 years. The predominance of women confirmed the expected demographics of PMBCL. After the iPET/CT evaluation, 62 patients (68.9%) had a positive iPET/CT (D4-5). Table 2 summarizes the population and lymphoma characteristics of interim PET positive and negative groups. Both patient groups were mostly well balanced (Table 2).

**Table 2:** Baseline characteristics of analyzed PMBCL patients.

Features	iPET negative (*n* = 28)	iPET positive(*n* = 62)	*P*- value
Median age, years (range)	38 (19–61)	36 (20–67)	0.579
Sex			0.747
male	10	20	
female	18	42	
B symptom			0.069
Yes	10	35	
No	18	27	
LDH			0.536
Elevated	23	54	
Normal	5	8	
ECOG			0.948
0	10	19	
1	13	31	
2	3	6	
3	2	6	
Extranodal involvement			0.887
Yes	14	32	
No	14	30	
Bone marrow involvement			0.503
Yes	0	1	
No	27	60	
IPI			0.629
N/A	0	1	
Low	15	33	
Low intermediate	7	18	
High intermediate	3	8	
High	3	2	
Metric PET analyzed subgroup	iPET negative (*n* = 11)	iPET positive (*n* = 24)	
MTV (mean, cm3)	242	198	0.901
SUVmax (mean)	20	22	0.547
TLG (mean)	2634	2797	0.695

LDH: Lactate dehydrogenase; ECOG: Eastern Cooperative Oncology Group; IPI: International Prognostic Index, SUVmax: maximum standardized uptake value, MTV: metabolic tumor volume, TLG: total lesion glycolysis.

### Analysis of treatment response and survival

**Fig. 3:**
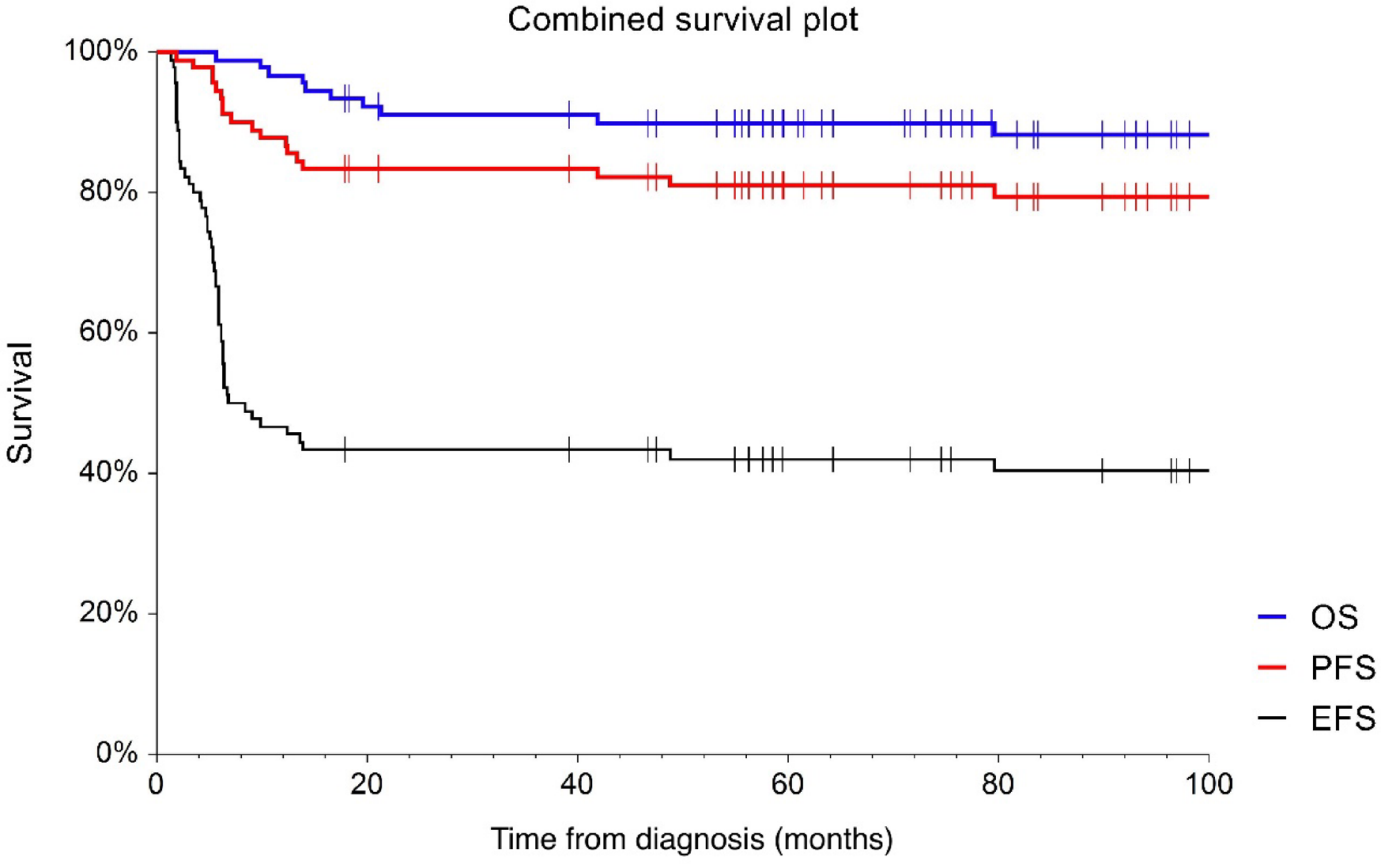
Combined survival plot showing overall survival (OS), progression free survival (PFS) and event free survival (EFS).

Overall, the treatment response according to final PET/CT was known in 87 (97%) of the patients. Of those, 52 patients (57.8%) achieved CR, 33 patients (36.7%) achieved partial remission, 5 patients (5,6%) was with progressive disease at the time of assessment. During the follow-up period, 15 patients (16.7%) progressed or relapsed and 10 patients (11.1%) died. Three-year OS (3-y OS) reached 91% (95% CI 0.85–0.97) and 3-year PFS (3-y PFS) 83.3% (95% CI 0.76–0.91) of the entire cohort. (Fig. 3)

**Fig. 4:**
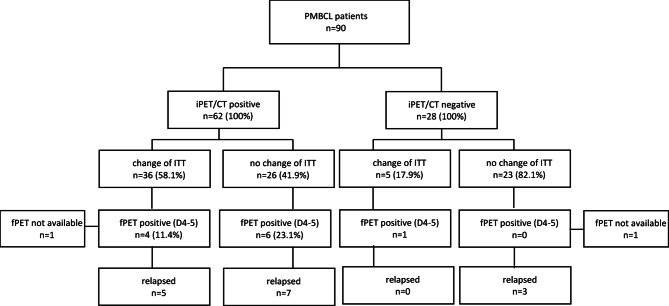
Diagram showing outcome of patients.

Subanalysis of the groups showed 3-y OS 96.2% in iPET/CT negative (D1-3) patients (*n* = 28) and 88.7% (*n* = 62) in iPET/CT positive (D4-5) patients (*p* = 0.18). The 3-year PFS in the iPET D1-3 group was 92.9% compared to 81.1% in the D4-5 group (*p* = 0.17). The negative predictive value (NPV) of iPET was 89.3%, while the positive predictive value (PPV) was only 19.4%. In addition, event free survival (EFS) was estimated, 3-y EFS was 75% and 29% in the iPET/CT negative and positive subgroups (*p* < 0.01). Of the iPET-positive patients (*n* = 62), therapy was changed in 36 cases, of whom 5 patients experienced progression or relapse. Among the 26 patients with positive iPET who did not undergo a therapy change, 7 patients experienced relapse or progression. (Fig. 4)

### Analysis of the metric PET/CT parameters

Scans of the all pts with iPET measurable disease were analyzed in terms of metric PET parameters (SUVmax, MTV and TLG) along with the change from the baseline PET (dSUVmax, dMTV and dTLG) in the pts from a single center (Olomouc, *n* = 49). The Youden index (maximum [sensitivity + specificity − 1]) was used to select the cut-off point that provided the best discrimination for event-free survival. Corresponding sensitivity and specificity values for each parameter are presented in Table 3.

**Table 3:** Optimal cut offs values for EFS event prediction.

	cut off value	AUC area	*P* value	Sensitivity	1 - Specificity	Specificity	SE + SP
iPET SUVmax	3.85	0.814	0.0004	0,750	0,105	0,895	1,645
iPET MTV	2.0	0.712	0.018	0,750	0,316	0,684	1,434
iPET TLG	7.5	0.721	0.014	0,750	0,316	0,684	1,434
dSUVmax	80%	0.897	< 0.0001	0,800	0,067	0,933	1,733
dMTV	92%	0.760	0.009	0,750	0,267	0,733	1,483
dTLG	98%	0.797	0.003	0,800	0,267	0,733	1,533

**Fig. 5:**
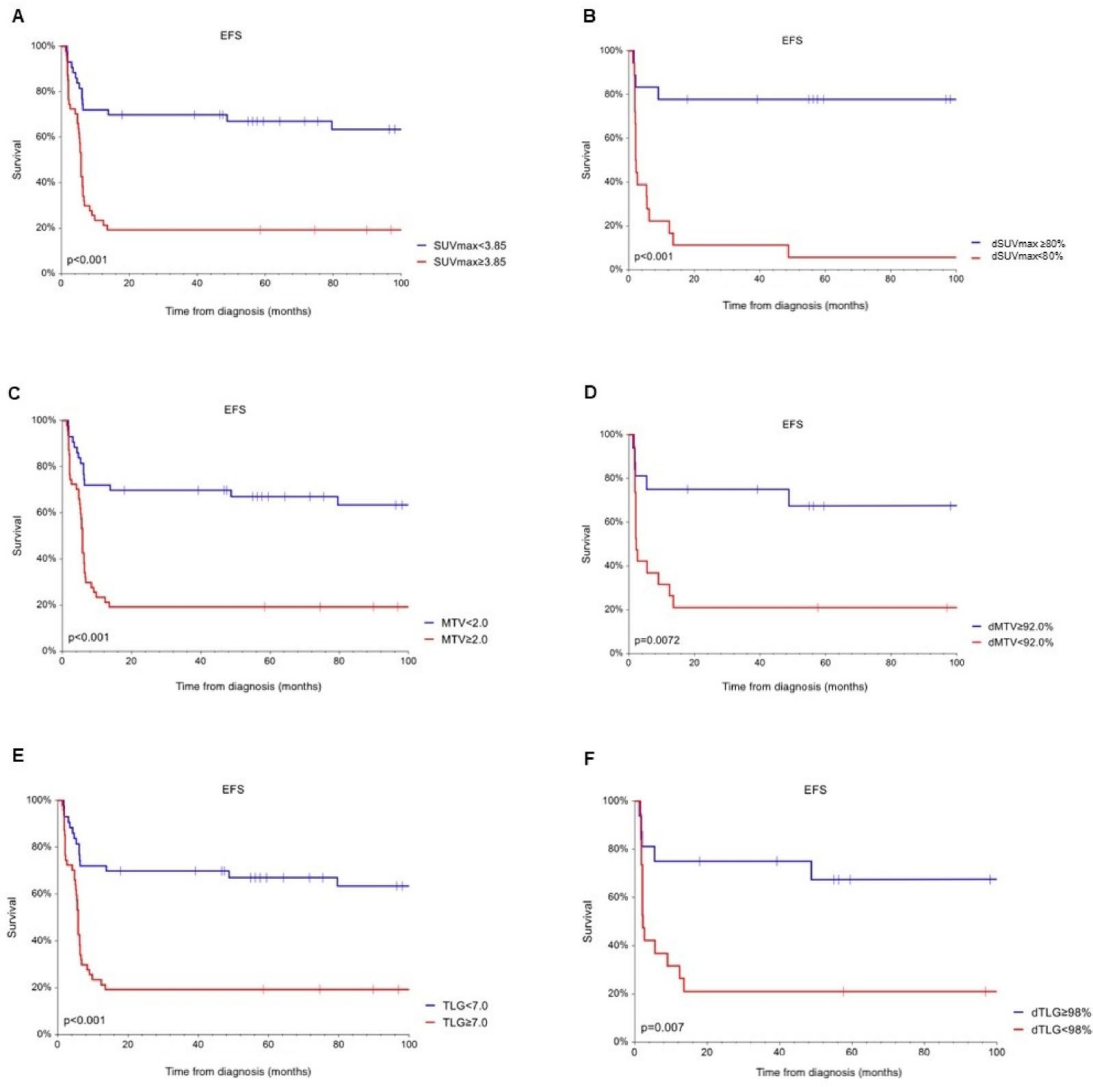
Event free survival of the patients according to relation to cut off values of metric PET parameters: (**A**) SUVmax, (**B**) dSUVmax, (**C**) MTV, (**D**) dMTV, (**E**) TLG, (**F**) dTLG.

3-y EFS of patients with SUVmax, MTV and TLG over estimated cut off values was 19.1%, compared to 69.8% for those below the cut off values (*p* < 0.01). Similar results were observed with PET/CT parameters reflecting the change, 3-y EFS of patients with dMTV and dTLG over the estimated cut off value was 75.0%, under the cut off 21.1% (*p* = 0.0072). 3-y EFS for dSUVmax was 77.8% and 11.1% (*p* < 0.01). In general, the outcome of patients with a change in analyzed values over the established cut off value was significantly more favorable. (Fig. 5)

## Discussion

An important challenge in the clinical management of PMBCL is the early identification of patients at high risk of therapy failure. Given that PMBCL patients are usually young age, the late toxicity associated with radiotherapy in this population can lead to severe, long-term complications and studies regarding omission of radiotherapy were conducted^23^. PET/CT imaging during the course of treatment is regarded as a potentially valuable tool, offering critical insights into relapse risk assessment^24^.

We have, to our knowledge, conducted the largest analysis of the interim PET/CT in PMBCL.

Our study showed a marginal - statistically insignificant - difference in survival based on the Deauville score in positive and negative iPET patients group, respectively, which is in accordance with published results^25,26^. This is mainly due to the very low positive predictive value of the interim PET (19.4%), which mirrors the results of many studies suggesting that PET/CT during or after the chemotherapy demonstrates positivity in 35–50% of patients with PMBCL^17^. The false positivity of iPET/CT in PMBCL might be caused by inflammatory behavior of the disease and the presence of residual fibrotic tissue in the mediastinal mass which might be confused with active tumor tissue. Another reason might be timing of the procedure or subjective interpretation of the scan and consolidation treatment applied to the interim PET + patients, alleviating its predictive power of capturing PFS events. Due to all these reasons we conducted a rigorous radiomics analysis of interim PETs, confirming that the positive predictive value of interim PET/CT can be improved through the use of quantitative radiomics. These measures provide objective assessment of tumor burden and its metabolic activity along with its change during the treatment. We have identified semiquantitative parameters measured such as maximum standardized uptake value (SUVmax), metabolic tumor volume (MTV) or total lesion glycolysis (TLG), which might enhance the predictive potential of iPET. We have analyzed event-free survival (EFS) to capture not only disease progression or relapse but also the physician’s decision to change therapy, considering the original intent-to-treat approach due to ineffectiveness.

Our study has several limitations. Due to the retrospective nature of the study, we were unable to perform histological verification to confirm PET/CT positivity in suspected cases. The information about primary intent-to-treat plan (ITT) might not be accurate in some patients, where it was recorded retrospectively. In our study, two different PET/CT systems were used, however, both systems were harmonized by EARL accreditation assuring the comparability of measured values. Another limitation is the heterogeneity in the timing of interim PET/CT scans, which were performed after the second, third, or, in a minority of cases, the fourth cycle of chemotherapy. Although this variability may influence PET results, it reflects real-world clinical practice and local institutional protocols, which often differ in the absence of universally adopted timing standards for iPET in PMBCL. Importantly, only four patients in our cohort underwent iPET after the fourth cycle, making the impact of this subgroup on the overall analysis negligible. Finally, we did not apply formal correction for multiple comparisons, such as the Bonferroni method, which could be considered when evaluating several semiquantitative PET/CT parameters. However, this study was partly exploratory in nature, aiming to identify potentially relevant radiomic metrics that may improve risk stratification in PMBCL. Applying overly stringent corrections at this stage might suppress promising associations that deserve further validation. Moreover, given the limited sample size (90 patients, with even smaller subgroups used for some analyses), correction could further reduce statistical power. Therefore, the results should be interpreted with caution and viewed as hypothesis-generating.The quality of the results of the analysis could be improved by the independent validation cohort.

In conclusion, visual analysis of the interim PET solely with the Deauville scale has its limitations, which might be overcome by incorporating the analysis of PET radiomic parameters. Future research should focus on refining iPET assessment with using the combination of qualitative and quantitative parameters, using large, independent cohorts and defining the role of the iPET as a surrogate endpoint in clinical practice. In addition, other clinical factors precising patient stratification and identifying chemoresistant individuals should be integrated. The most promising approach may involve blood sampling for detecting circulating tumor DNA^27,28^. Using iPET/CT as a prognostic tool and reliable factor in PET-guided therapy could be enhanced through additional prospective studies.

## Data Availability

The datasets analyzed in this study are available from the corresponding author upon reasonable request. The corresponding author had full access to all data in the study and takes responsibility for the integrity of the data and the accuracy of the data analysis.
